# Rapid Cure for a Cold

**Published:** 1880-06

**Authors:** 


### RAPID CURE FOR A COLD.

R. RUDOLF reports, in the *Gazetta Medicina Italiana*, the following observation made on himself. Being seized with a severe coryza, he happened to chew one or two twigs of eucalyptus, at the same time swallowing the saliva secreted, which had a bitter and aromatic flavor. To his surprise he found that, in the course of half an hour, the nasal catarrh had disappeared. Some days later he was seized with another attack from a fresh exposure to cold, when the same treatment followed by an equally fortunate result. He then prescribed the remedy to several of his patients, all of whom were benefited in the same way. He believes that this treatment is only suitable in acute cases.—*British Medical Journal, Jan, 24, 1880.*

